# Supplementation with crude rhubarb extract lessens liver inflammation and hepatic lipid accumulation in a model of acute alcohol-induced steato-hepatitis

**DOI:** 10.1186/2049-3258-72-S1-P6

**Published:** 2014-06-06

**Authors:** Audrey M Neyrinck, Usune Etxeberria, Alexandra Jouret, Nathalie M Delzenne

**Affiliations:** 1 Louvain Drug Research Institute, Metabolism and Nutrition Research Group, Université catholique de Louvain, Brussels, Belgium

## Background

Binge consumption of alcohol is an alarming global health problem and is implicated in the pathophysiology of alcoholic liver disease (ALD) [1]. In its early stages, ALD it is characterized by fatty liver [1]. Moreover, numerous studies are supporting the concept that beyond this hepatic steatosis physiological processes, inflammation occurred in the progression of the disease [2]. Strategies directed to reduce fat accumulation and local hepatic inflammation caused by alcohol consumption might succeed blocking the evolution of ALD. In this sense, natural plants rich in bioactive constituents are attracting a growing interest as new therapeutic agents. Several studies have reported health benefits coming from rhubarb extract rich in anthraquinones [3,4]. The aim of the present study was to test the potential hepatoprotective effects of rhubarb extract supplementation in a model of acute alcohol-induced steato-hepatitis.

## Materials and methods

Male C57Bl6J mice were fed with a control diet supplemented or not with 0.3% rhubarb extract (Laboratoires Ortis, Belgium). After 17 days, mice received a huge dose of ethanol (6g/kg bw) and were sacrificed 6 hours after the alcohol challenge. Liver oil red O staining and hepatic lipid contents were determined to assess hepatic steatosis whereas the expression of pro-inflammatory markers were analysed in the liver tissue by quantitative PCR to assess hepatic inflammation.

## Results

**Figure 1:**
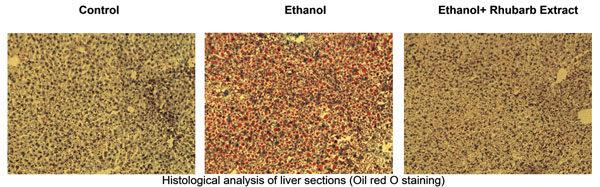
Fat accumulation in the liver

**Figure 2:**
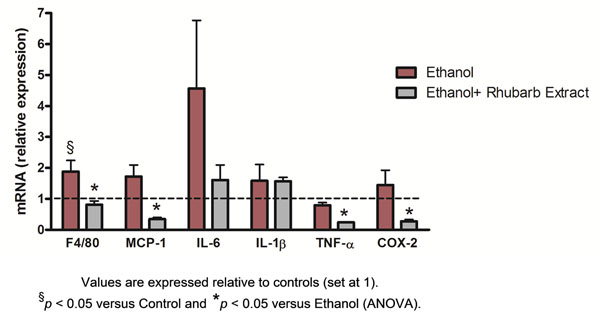
Expression of hepatic inflammatory markers

Ethanol administration caused a massive increase of hepatic triglycerides and, in a lesser extent, in total cholesterol inside the liver tissue (table 1). Rhubarb extract decreased hepatic triglyceride content. This effect was confirmed by histological analysis (figure 1). Interestingly, rhubarb extract supplementation blunted the ethanol-induced F4/80 expression, suggesting a lower recruitment of inflammatory cells inside the liver (figure 2). Moreover, proinflammatory markers such as TNF-α, IL-6, MCP-1 and COX-2 were down-regulated in the liver of mice fed with rhubarb extract (figure 2).

**Table 1:** Hepatic lipid contents

	Control	Ethanol	Ethanol+ Rhubarb Extract
**(nmol/mg prot)**			
Triglycerides	233.60 ± 18.64^a^	454.40 ± 55.65^b^	345.60 ± 32.24^a,b^
Total cholesterol	90.38 ± 8.62	161.50 ± 33.06	108.50 ± 14.45

Data with different superscript letters are significantly different at *p*<0.05 (ANOVA)

## Conclusions

These results suggest that rhubarb extract rich in anthraquinones might decrease liver tissue injury, namely hepatic lipid accumulation and inflammatory disorders caused by acute alcohol consumption.

## References

[B1] Shukla SD Pruett SB Szabo G Arteel GE Binge ethanol and liver: new molecular developments Alcoholism, clinical and experimental research 2013 37 4 550 557 10.1111/acer.12011 PMC4831914 23347137

[B2] Huang LL Wan JB Wang B He CW Ma H Li TW Kang JX Suppression of acute ethanol-induced hepatic steatosis by docosahexaenoic acid is associated with downregulation of stearoyl-CoA desaturase 1 and inflammatory cytokines Prostaglandins, leukotrienes, and essential fatty acids 2013 88 5 347 353 10.1016/j.plefa.2013.02.002 23474173

[B3] Moon MK Kang DG Lee JK Kim JS Lee HS Vasodilatory and anti-inflammatory effects of the aqueous extract of rhubarb via a NO-cGMP pathway Life sciences 2006 78 14 1550 1557 16269157 10.1016/j.lfs.2005.07.028

[B4] Raal A Pokk P Arend A Aunapuu M Jogi J Okva K Pussa T Trans-resveratrol alone and hydroxystilbenes of rhubarb (Rheum rhaponticum L.) root reduce liver damage induced by chronic ethanol administration: a comparative study in mice Phytotherapy research : PTR 2009 23 4 525 532 19067386 10.1002/ptr.2665

